# Slow Replication Fork Velocity of Homologous Recombination-Defective Cells Results from Endogenous Oxidative Stress

**DOI:** 10.1371/journal.pgen.1006007

**Published:** 2016-05-02

**Authors:** Therese Wilhelm, Sandrine Ragu, Indiana Magdalou, Christelle Machon, Elodie Dardillac, Hervé Técher, Jérôme Guitton, Michelle Debatisse, Bernard S. Lopez

**Affiliations:** 1 CNRS UMR 8200, Gustave Roussy Cancer Institute, Université Paris-Saclay, Team labeled “Ligue 2014”, Villejuif, France; 2 Laboratoire de Biochimie et Toxicologie, Hospices Civils de Lyon, Centre Hospitalier Lyon-Sud, Pierre-Bénite, France; 3 Laboratoire de Chimie Analytique, Université de Lyon, Université Lyon 1, ISPB Faculté de Pharmacie, Lyon, France; 4 Institut Curie, Centre de Recherche, Paris, France, UPMC Université Paris 06, Paris, France, CNRS UMR 3244, Paris, France; 5 Laboratoire de Toxicologie, Université Lyon 1, ISPB, Faculté de Pharmacie, Lyon, France; University of Washington School of Medicine, UNITED STATES

## Abstract

Replications forks are routinely hindered by different endogenous stresses. Because homologous recombination plays a pivotal role in the reactivation of arrested replication forks, defects in homologous recombination reveal the initial endogenous stress(es). Homologous recombination-defective cells consistently exhibit a spontaneously reduced replication speed, leading to mitotic extra centrosomes. Here, we identify oxidative stress as a major endogenous source of replication speed deceleration in homologous recombination-defective cells. The treatment of homologous recombination-defective cells with the antioxidant N-acetyl-cysteine or the maintenance of the cells at low O_2_ levels (3%) rescues both the replication fork speed, as monitored by single-molecule analysis (molecular combing), and the associated mitotic extra centrosome frequency. Reciprocally, the exposure of wild-type cells to H_2_O_2_ reduces the replication fork speed and generates mitotic extra centrosomes. Supplying deoxynucleotide precursors to H_2_O_2_-exposed cells rescued the replication speed. Remarkably, treatment with N-acetyl-cysteine strongly expanded the nucleotide pool, accounting for the replication speed rescue. Remarkably, homologous recombination-defective cells exhibit a high level of endogenous reactive oxygen species. Consistently, homologous recombination-defective cells accumulate spontaneous γH2AX or XRCC1 foci that are abolished by treatment with N-acetyl-cysteine or maintenance at 3% O_2_. Finally, oxidative stress stimulated homologous recombination, which is suppressed by supplying deoxynucleotide precursors. Therefore, the cellular redox status strongly impacts genome duplication and transmission. Oxidative stress should generate replication stress through different mechanisms, including DNA damage and nucleotide pool imbalance. These data highlight the intricacy of endogenous replication and oxidative stresses, which are both evoked during tumorigenesis and senescence initiation, and emphasize the importance of homologous recombination as a barrier against spontaneous genetic instability triggered by the endogenous oxidative/replication stress axis.

## Author Summary

Endogenous stress is an important stress because it challenges cells daily. However, endogenous stress is difficult to apprehend. Replication forks are routinely hindered by different endogenous stresses. Because homologous recombination plays a pivotal role in the reactivation of arrested replication forks, defects in homologous recombination reveal the initial endogenous stress(es). Here we identify endogenous oxidative stress among the different potential endogenous stresses as being responsible for spontaneous replication defects in homologous recombination-defective cells. Therefore, oxidative and replication stresses, which are both evoked during tumorigenesis and senescence initiation, are linked, and homologous recombination acts as a barrier against spontaneous genetic instability triggered by endogenous oxidative/replication stress.

## Introduction

The maintenance of genome stability relies on successful DNA replication and equal partitioning of the duplicated DNA during mitosis. However, stresses of endogenous or exogenous origin can jeopardize genome stability, fueling cancer development or senescence. Endogenous stress is a significant biological phenomenon because cells are chronically exposed to such stress throughout their lifespan; thus, endogenous stress constitutes a major source of genome instability. In this context, replication stress and oxidative stress (OS) are two major endogenous stresses that are frequently proposed as primary sources of genome instability.

The progression of replication forks (RFs) is routinely obstructed by obstacles of endogenous and exogenous origin on the DNA, leading to the stalling, collapse or breakage of RFs and genome instability [1,2]. Such hindrances to fork progression also challenge the completion of DNA replication, resulting in unrepaired/unreplicated DNA at mitotic entry and thus mitotic defects and aneuploidy. Notably, replication failure leads to mitotic anaphase bridges and breaks at common fragile sites [1–3]. Moreover, although they do not contain DNA, active mitotic extra centrosomes (MECs) are a consequence of replication stress, leading to multipolar chromosomal segregation or chromosome lagging, thus amplifying chromosome instability from a local problem during replication to a genome-wide problem after mitosis [4]. Consistently, spontaneous activation of the DNA damage response (DDR) has been described as a consequence of endogenous DNA replication stress in pre-cancerous cells and during the early stages of malignancy or senescence [5–8]. Similarly, centrosome abnormalities have also been described during the early stages of malignancy [9–11], lending molecular support to the theory that tumors have a clonal origin and arise through multipolar chromosomal segregation, which was proposed by Boveri one century ago [12,13]. Therefore, elucidating the origin of the endogenous factors that are responsible for spontaneous replication stress is of crucial importance. However, little is known regarding the nature of endogenous replication stress because this phenomenon is difficult to detect and analyze.

Homologous recombination (HR) is an evolutionarily conserved process that plays a pivotal role in the protection and reactivation of blocked RFs and in the repair of replication-associated double-strand breaks [1,2,14]. In addition, actors in the HR process can also protect the arrested replication forks, even without leading to an HR outcome [15–17]. Since Because RF progression is routinely arrested by endogenous stress, an HR deficiency should reveal the endogenous replication stress. Supporting this conclusion is the fact that unchallenged HR-deficient (*HR*^*-*^) cells spontaneously display lower rates of RF progression [4,18]. Importantly, the endogenous replication defect in *HR*^*-*^ cells leads to the formation of MECs and unbalanced multipolar chromosomal segregation [4]. Therefore, identifying the causes of endogenous stress that lead to RF deceleration in *HR*^*-*^ cells is essential for deciphering the process(es) responsible for spontaneous chromosome instability in general.

There are multiple causes of endogenous replication stress that are difficult to unify [19]. Because OS is an important chronic endogenous stress, in this study, we addressed whether OS, as a possible endogenous stress, may be responsible for the spontaneous deceleration of RFs in *HR*^*-*^ cells. Endogenous OS results from the production of reactive oxygen species (ROS) as by-products of cellular metabolism. The most well-documented process by which OS generates genome instability is mutagenesis through DNA injuries caused by the generation of oxidized bases and apurinic sites. Note that *in vitro*, some oxidative DNA damage is able to impede the progression of polymerases [20]. In addition, DNA double-strand breaks (DSBs) generated by OS can also provoke replication fork collapse. In addition to DNA damage, OS can affect replication through additional processes, such as by regulating the activities of replication proteins [21–26]. Finally, many cells that are affected by the DNA damage response (DDR), such as ataxia telangiectasia, Fanconi anemia, Cockayne syndrome or Bloom syndrome cells, or defective in BRCA1 exhibit elevated levels of endogenous ROS [27–32]. Here, we address whether OS might control the slow replication velocity of *HR*^*-*^ cells.

We show that OS can affect the nucleotide pool and replication fork progression and generate MEC. In *HR*^*-*^ cells, we revealed that endogenous OS is a major source of endogenous replication stress. These data show that the two most typically evoked endogenous stresses, OS and replication stresses, are linked at the origin of spontaneous genome instability. The data also emphasize the importance of HR as a barrier against spontaneous genetic instability triggered by the endogenous oxidative/replication stress axis.

## Results

### N-acetyl-cysteine or maintaining low oxygen levels rescues the replication fork speed in *HR*^*-*^ cells

The cell lines used in our study were derived from hamster V79 cells (described in Table 1 and in the Materials and Methods) because several extensively characterized *HR*^*-*^ cell lines exist in this background. Here we used cell lines with a loss-of-function mutation in the endogenous *BRCA2* gene (V-C8 cells) [33,34] or cell lines expressing a dominant-negative form of RAD51 (V79SM24), which specifically inhibits gene conversion [35,36].

**Table 1:** Cell lines and derivatives.

*Cell lines*	*Origin*	*Plasmid or chromosome transferred*
V79	Wild-type hamster cell line	None
V79puro	V79	Empty expression vector (pCDNA3-puro) [36]
V79SM24 (HR^-^)	V79	Vector coding for dominant-negative SMRad51 (pCDNA3-puro) [36]
VC-8 (HR^-^)	V79-deficient in *Brca2* [33]	None
VC-8#13	VC-8 complemented [33]	Human *BRCA2* is expressed from human chromosome 13

**Fig 1:**
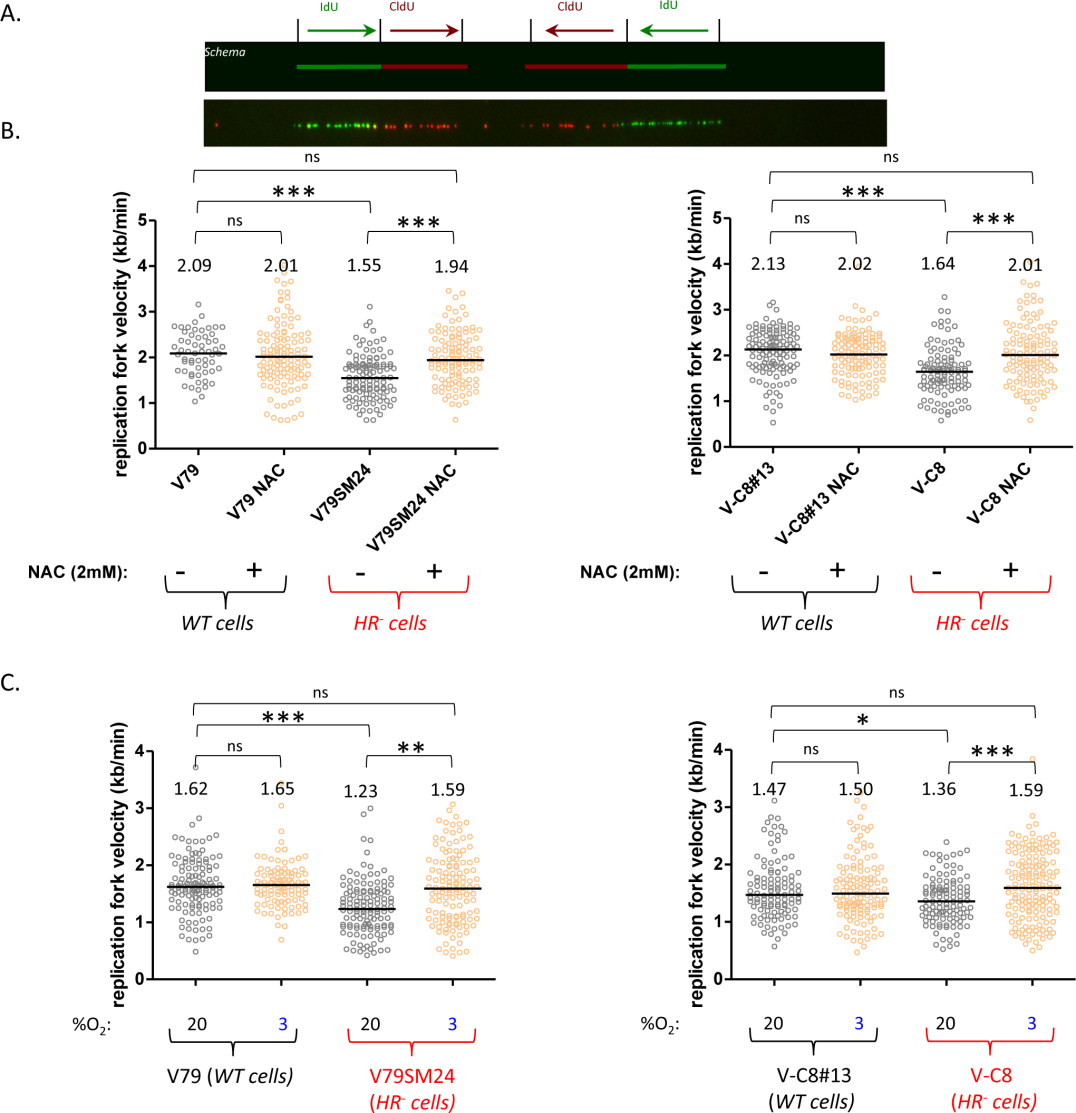
Impact of NAC or low O_2_ levels on the genome-wide replication fork speed. **A/** Examples of combed DNA fibers with replication tracts: IdU (green), CldU (red) and the scheme of the experiment (upper panels). **B**/ RF speed distribution in V79 cells and derivatives (left) and V-C8 cells and derivatives (right) after exposure to NAC (3 mM). **C**/ RF speed distribution in V79 cells and derivatives (left) and V-C8 cells and derivatives (right) that were maintained at 20% versus 3% O_2_. The median and p-values are indicated in the histogram (*P < 0.05; **P < 0.01; ***P < 0.001). The median values are represented as horizontal black lines. ns: not significant. Approximately 100–120 fibers were scored per condition.

The RF speed was monitored using single-molecule analysis via molecular combing (Fig 1A). As described previously, *HR*^*-*^ cells (V79SM24 and V-C8) experience significantly slower RF speeds than control cells (Fig 1B and 1C) [4,18]. Note that silencing the pivotal HR protein RAD51 also leads to a significant deceleration of the replication speed in human JEFF cells (S1 Data). To address whether endogenous OS causes a slow replication fork speed in HR-deficient cells, we first treated the cells with the antioxidant N-acetyl-cysteine (NAC). Interestingly, while exposure to NAC did not influence the RF kinetics of wild-type (WT) cells, this treatment increased the RF speed of *HR*^*-*^ cells to the levels observed in their respective controls (Fig 1B).

To corroborate the impact of endogenous OS on the RF speed, we then maintained the cells at low O_2_ level (3%) for several weeks. Under these conditions, the RF kinetics of the WT cells remained unaffected, whereas the RF speed in *HR*^*-*^ cells increased to levels comparable to those observed in the WT cells (Fig 1C). Taken together, these data show that the slow replication fork progression in HR-deficient cells can be alleviated by decreasing endogenous OS.

### N-acetyl-cysteine or maintaining low oxygen levels reduces MECs

**Fig 2:**
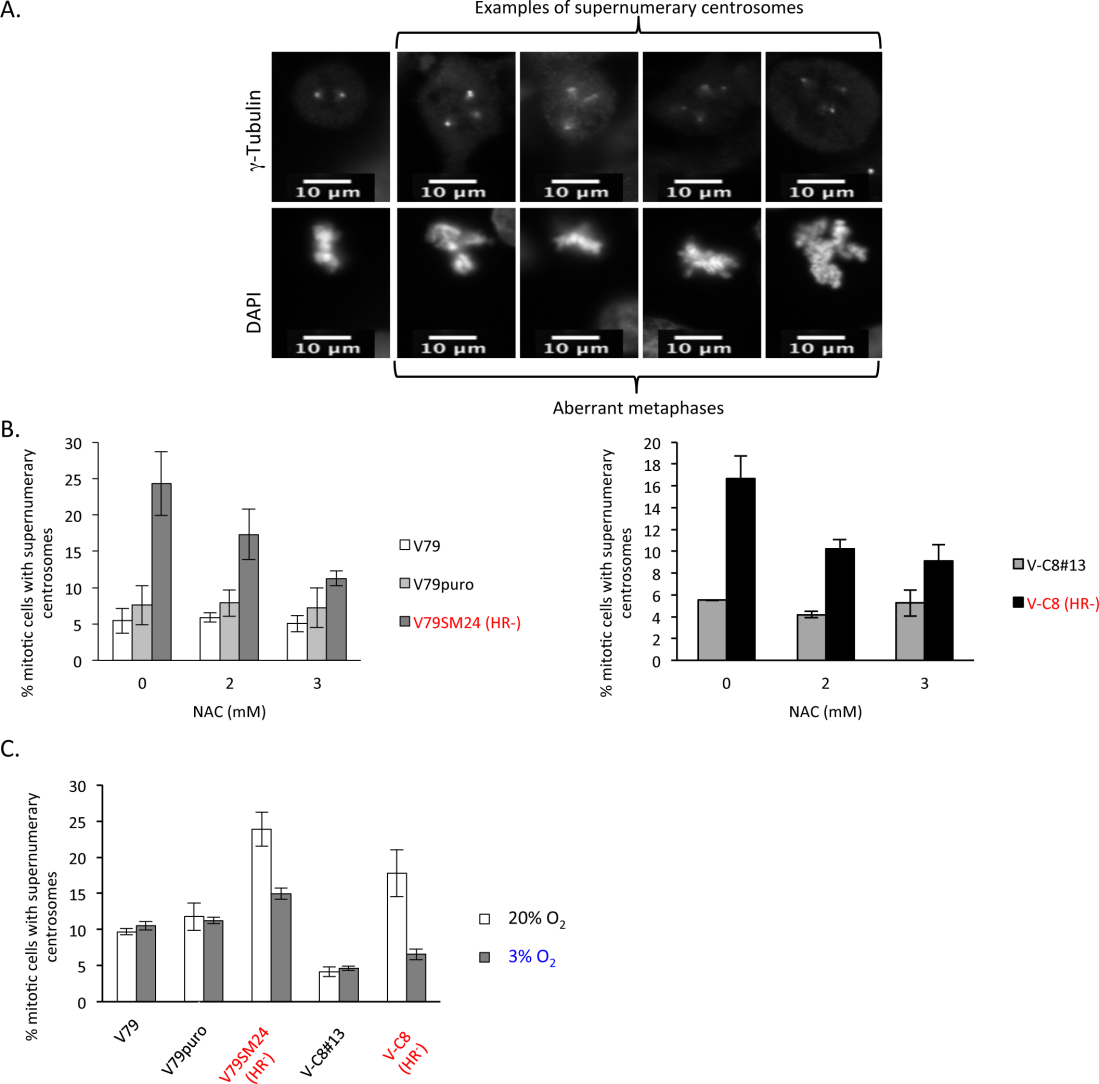
Impact of NAC or low O_2_ levels on supernumerary centrosomes in mitotic cells (MEC). **A/** Examples of labeled centrosomes in mitotic cells (see chromosomal DAPI staining in lower panels). Left photograph: normal centrosome number (= 2); right photographs: aberrant centrosome numbers (≠2) leading to metaphase alterations (see DNA labeling in lower panels). Scale bar, 10 μm. **B/** Frequencies of mitotic cells with aberrant centrosome numbers. Left histograms: V79 cells and derivatives; right histograms: V-C8 cells and derivatives. **C/** Impacts of a low level of O_2_ (3%) on MECs. The mean value +/- s.d. was calculated from at least three independent experiments. In total, 150 mitoses were scored for each experiment and condition.

MECs have been shown to be tractable predictive markers of mitotic dysfunction in response to low or endogenous replication stress [4]. Thus, we measured the occurrence of MECs (Fig 2A) after NAC treatment or in cells cultured at 3% oxygen.

In unchallenged conditions, the frequency of MECs is spontaneously higher in *HR*^*-*^ cells than in WT cells, as described previously [4,35,37]. While treatment with NAC did not affect the frequency of MECs in the WT cells, this treatment decreased the frequency of MECs in *HR*^*-*^ cells (Fig 2B). Consistently, maintaining the cells at 3% O_2_ recovered the frequency of MECs in *HR*^*-*^ cells without altering this frequency in WT cells (Fig 2C).

Taken together, these data show that endogenous oxidative stress accounts for the spontaneous MECs that occur in HR-deficient cells.

### HR-deficient cells exhibit spontaneously higher levels of endogenous ROS

**Fig 3:**
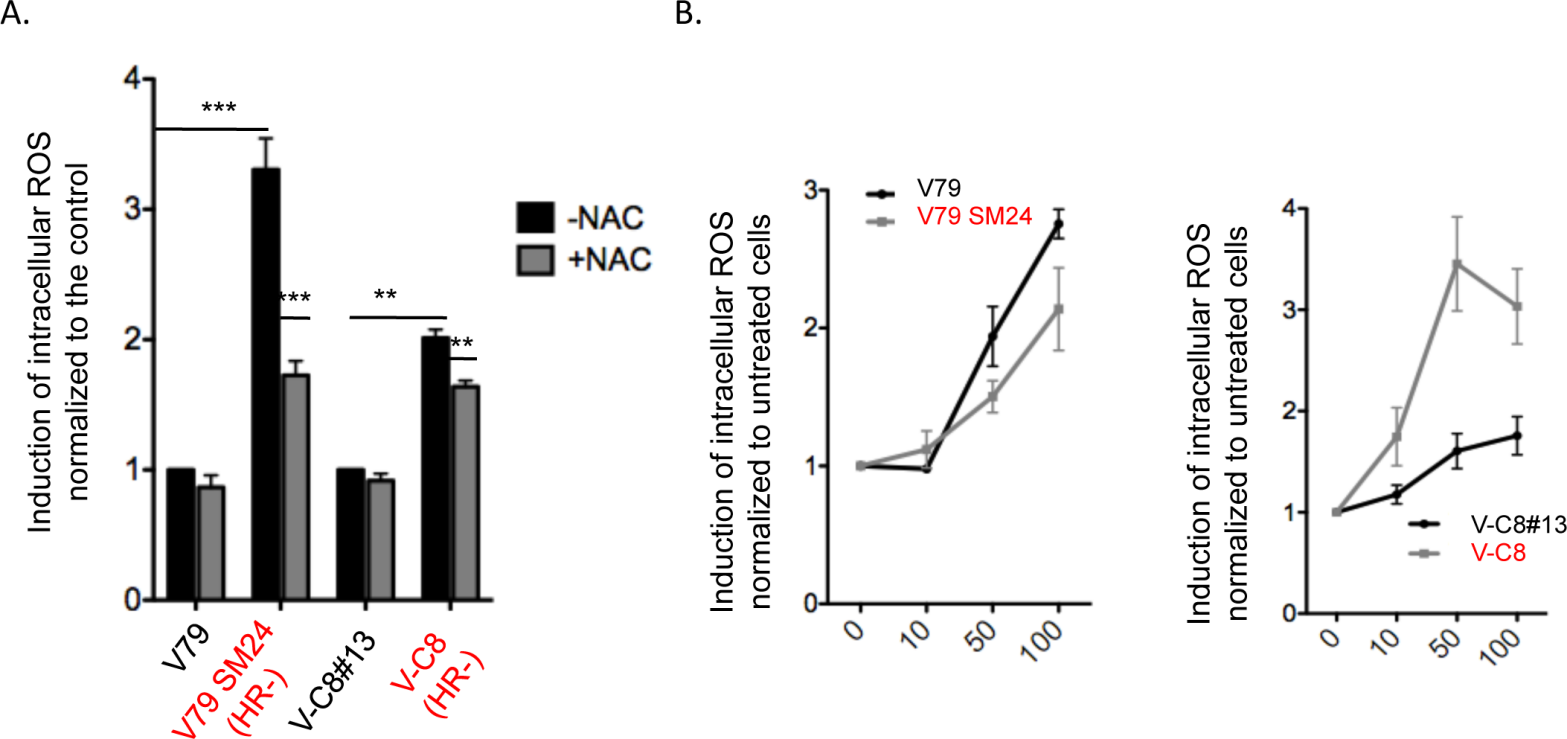
Level of intracellular ROS. **A/** endogenous ROS. NAC: 2mM. The mean value +/- s.d. was calculated from at least three independent experiments. **B/ After exposure to H**_**2**_**O**_**2**_. The value +/- s.d. was calculated from at least three independent experiments.

High levels of endogenous ROS have been described in different cells affected in the DDR [27–32]. More specifically, cells defective in BRCA1 exhibit high levels of endogenous ROS [27]. Therefore, although the underlying mechanisms remain puzzling, we tested the endogenous level of ROS in *HR*^*-*^ cells using the fluorescent probe 2’,7’-dichlorofluorescein diacetate (Fig 3). Similar to other DDR-defective cells, *HR*^*-*^ cells show higher levels of spontaneous ROS than their respective controls (Fig 3A). Treatment with NAC partially decreased the level of endogenous ROS in both WT and *HR*^*-*^ cells, but the levels remained higher in *HR*^*-*^ cells (Fig 3A).

The level of spontaneous intracellular ROS in *HR*^*-*^ cells corresponds to that of WT cells exposed to doses of H_2_O_2_ between 10 and 50 μM (Fig 3B). Exposure to H_2_O_2_ increased the intracellular level of ROS in both WT and *HR*^*-*^ cells, showing that ROS were not present at a saturated concentration in *HR*^*-*^ cells (Fig 3B).

Because OS might affect the replication speed (via different targets), the high level of endogenous ROS could account for the RF deceleration observed in *HR*^*-*^ cells. However, it is essential to verify that increasing intracellular ROS levels actually affect the replication speed and MECs.

### H_2_O_2_ reduces the genome-wide replication speed and generates MECs

**Fig 4:**
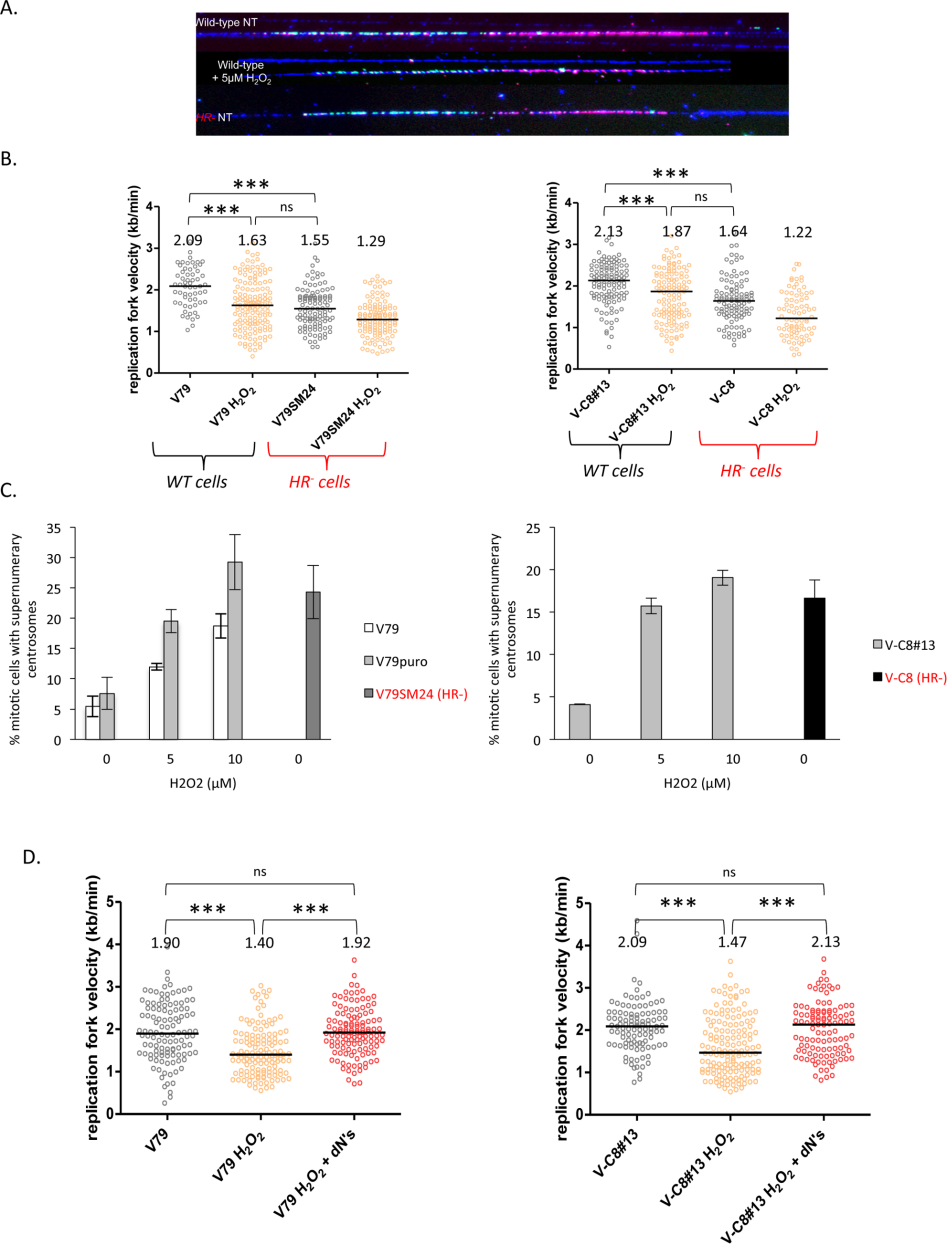
Impact of H_2_O_2_ on the genome-wide replication speed. **A/** Examples of molecular combing fibers. **B/** RF speed distribution in V79 cells and derivatives (left) and V-C8 cells and derivatives (right) after exposure to H_2_O_2_ (5 μM). The median values are represented as horizontal black lines. p-value: *P < 0.05; **P < 0.01; ***P < 0.001; ns: not significant. Approximately 100–120 fibers were scored per condition. **C/** Impact of H_2_O_2_ on MECs in V79 cells and derivatives (left) and V-C8 cells and derivatives (right). The mean value +/- s.d. was calculated from at least three independent experiments. In total, 150 mitoses were scored for each experiment and condition. **D/** The effects of the addition of dNs on the replication fork speed after exposure to H_2_O_2_. The replication fork speed distribution in V79 cells and derivatives (left) and V-C8 cells and derivatives (right) is presented. The numbers correspond to the median replication speed. The median (black lines) and p-values are indicated in the histogram (*P < 0.05; **P < 0.01; ***P < 0.001). ns: not significant. 100 to 145 fibers were scored per condition.

To sustain the impact of OS on replication fork progression, we treated WT cells with H_2_O_2_. The exposure of WT cells to 10 μM H_2_O_2_, which does not significantly affect the viability of WT or *HR*^*-*^ cells (S2 Data) led to levels of RF deceleration that were comparable to those observed in untreated *HR*^*-*^ cells (Fig 4A and 4B). H_2_O_2_ also decreased the replication speed in *HR*^*-*^ cells (Fig 4B), which is consistent with the increase in ROS (see Fig 3).

Importantly, under these conditions, MECs occurred at a frequency similar to that observed in unchallenged *HR*^*-*^ cells (compare Fig 4C). These data show that sub-lethal doses of H_2_O_2_ actually lead to the genome-wide deceleration of replication forks in association with MECs. Consistent with our data, exposure to the pro-oxidant agent H_2_O_2_ has been shown to activate the phosphorylation of Chk1 [38,39].

In various situations, reduced RF rates have been corrected by adding dNs to the culture medium [4,40–43]. More specifically, supplying dNs has been shown to correct the slow replication speed of the *HR*^*-*^ cells used here [4]. Consistent with these reports, the RF speed deceleration that occurs in response to OS can be alleviated by supplying dNs to the culture medium (Fig 4D).

### H_2_O_2_ alters the nucleotide pool

Among the different ways that OS might affect replication, the above results suggest that an alteration of the nucleotide pool might be a major cause of the OS-induced replication deceleration. Moreover, in the yeast *Saccharomyces cerevisiae*, the ribonucleotide reductase is oxidized by ROS [44]. Therefore, we analyzed the nucleotide pools.

**Fig 5:**
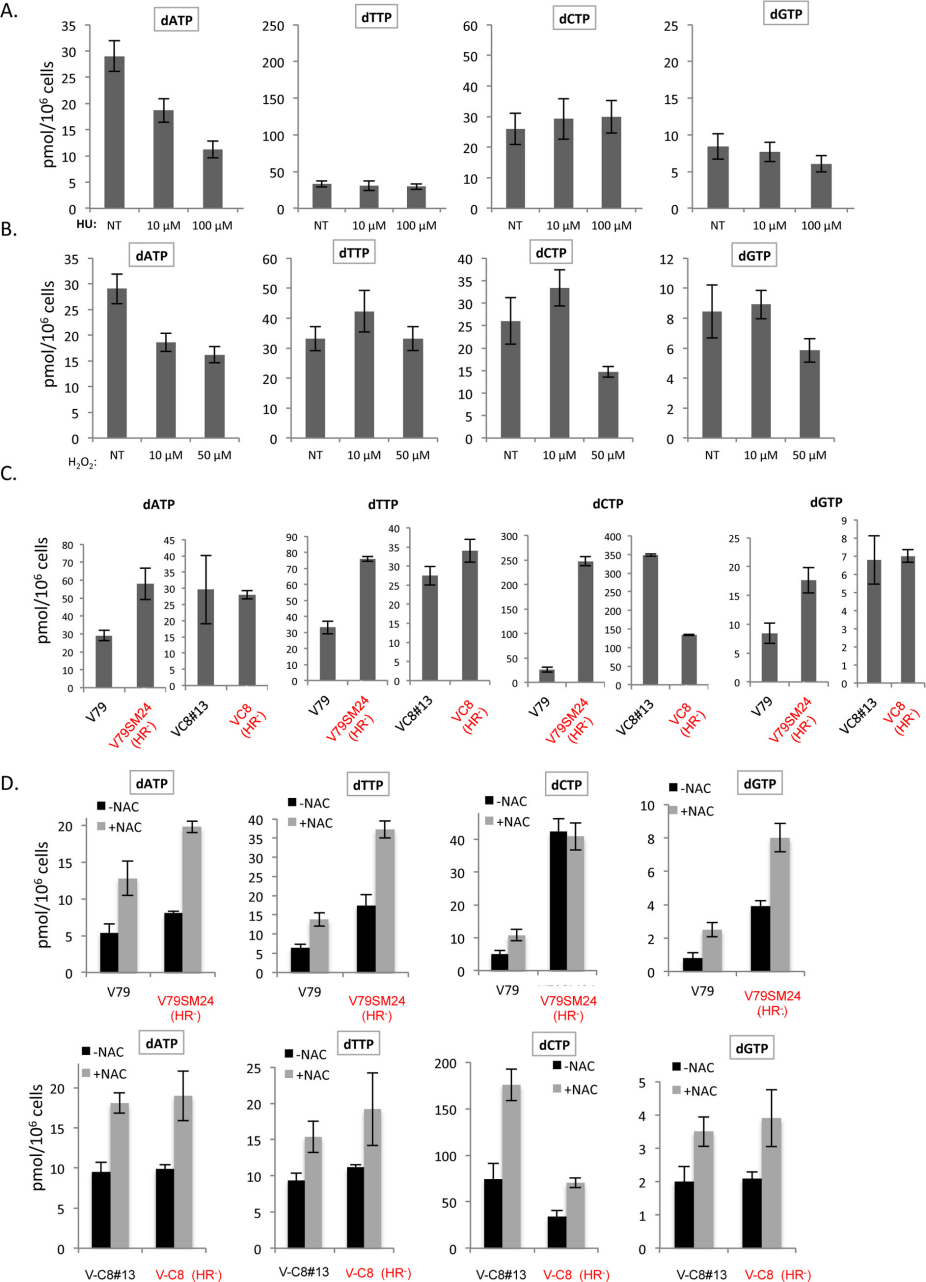
dNTP pool measurements. **A/** dNTP concentrations after exposure to different doses of HU. **B/** dNTP concentrations after exposure to H_2_O_2_. **C/** dNTP concentrations in unchallenged WT and *HR*^*-*^ cells. **D/** Impact of NAC (2 mM, 48 h) on dNTP concentrations in WT and *HR*^*-*^ cells. The experiments were performed in triplicate. NT: not treated.

First, we confirmed that the method [45] used here is actually able to detect subtle imbalances in the dNTP pool after exposure to low doses of HU. In particular, HU provoked a dose-dependent decrease in the dATP concentration (Fig 5A). Interestingly, the method was sensitive enough to detect the effect of HU at doses as low as 10 μM (Fig 5A). Exposure to H_2_O_2_ also led to an imbalanced dNTP pool (Fig 5B). Specifically, the dATP pool was affected at levels similar to those that result from exposure to 10 μM HU (compare Fig 5A and 5B). Consistently, exposure to 10 μM H_2_O_2_ also resulted in a slight oxidation of the ribonucleotide reductase subunit RRM2 (S3 Data). *HR*^*-*^ cells exhibit a spontaneously unbalanced dNTP pool in comparison to their respective controls, although the patterns are different in the two different kinds of HR-defective cells (Fig 5C).

Importantly, treatment with NAC increased the concentrations of the dNTP pools in both WT and *HR*^*-*^ cells (Fig 5D). These results are consistent with those obtained after measuring the replication speed. Indeed, both NAC and the dNs supply affect the replication speed similarly; both rescue the replication speed in *HR*^*-*^ cells without affecting the replication speed in WT cells.

In yeast, the nucleotide pool is a limiting factor for replication progression [46]. In mammalian cells, the fact that doses of HU as low as 10 μM, which cause only a slight imbalance in the dNTP pool (see Fig 5A), are able to affect the replication speed suggest that the dNTP pool is a limiting factor. Remarkably, the present data suggest that the dNTP pool might also be a limiting factor at 20% O_2_ but they also suggest that the dNTP pool might not be a limiting factor at low endogenous ROS level.

The above results concerning the dNTP pool can account for the rescue of the replication speed of *HR*^*-*^ cells by anti-oxidant treatments. However, these results do not exclude the possibility that other mechanisms also participate in the observed difference in replication speeds between unchallenged WT and *HR*^*-*^ cells maintained at 20% O_2_. Importantly, OS can also generate DNA damage that might more or less directly require HR to be processed. Indeed, BRCA1 and BRCA2 protect against double-strand breaks induced by oxidative DNA damage during DNA replication [47]. Therefore, OS-induced DNA breaks should accumulate in unchallenged *HR*^*-*^ cells, both due to the repair defect of OS-induced breaks and due to the higher level of endogenous ROS. Reciprocally, OS should induce HR. Indeed, both DSBs and replication stress stimulate HR [48,49]. Thus, we first measured the spontaneous accumulation of foci of the DNA break recognition proteins γH2AX and XRCC1 in *HR*^*-*^ cells. Second, we measured whether OS can actually induce HR.

### *HR*^*-*^ cells accumulate spontaneous γH2AX and XRCC1 foci that are rescued by antioxidant treatments

**Fig 6:**
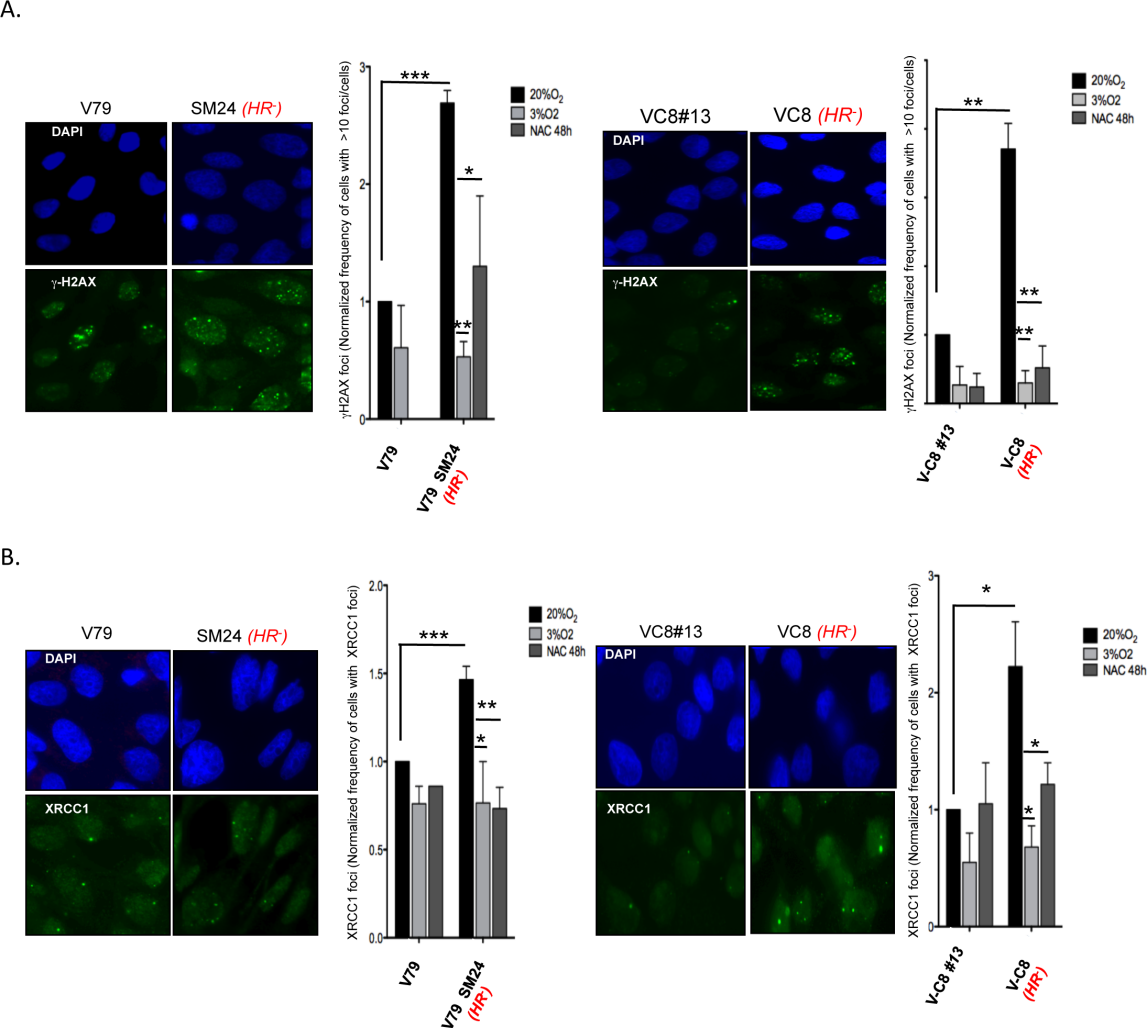
Spontaneous γH2AX and XRCC1 foci. **A/γ**H2AX foci. Left panels: example of γH2AX foci. The nuclei are counterstained with DAPI (blue). Right panels: normalized frequency of cells with >10 spontaneous foci. **B/** XRCC1 foci. Left panel: example of XRCC1 foci. The nuclei are counterstained with DAPI (blue). Right panel: normalized frequency of cells with >10 spontaneous foci. At least 200 cells were counted. NAC: 2 mM, 48 h. The data were obtained from three independent experiments (error bars: s.e.m.).

Using specific antibodies, we analyzed via immunofluorescence the spontaneous accumulation of foci of the γH2AX and XRCC1 proteins, which recognize double- and single-strand breaks, respectively (Fig 6).

Interestingly, *HR*^*-*^ cells exhibited spontaneously higher levels of both γH2AX and XRCC1 foci (Fig 6A and 6B). Importantly, increased foci were suppressed both by treatment with NAC and by maintaining the cells at 3% O_2_ (Fig 6A and 6B), showing that these types of damage actually resulted from endogenous ROS.

### H_2_O_2_ stimulates intrachromosomal HR

**Fig 7:**
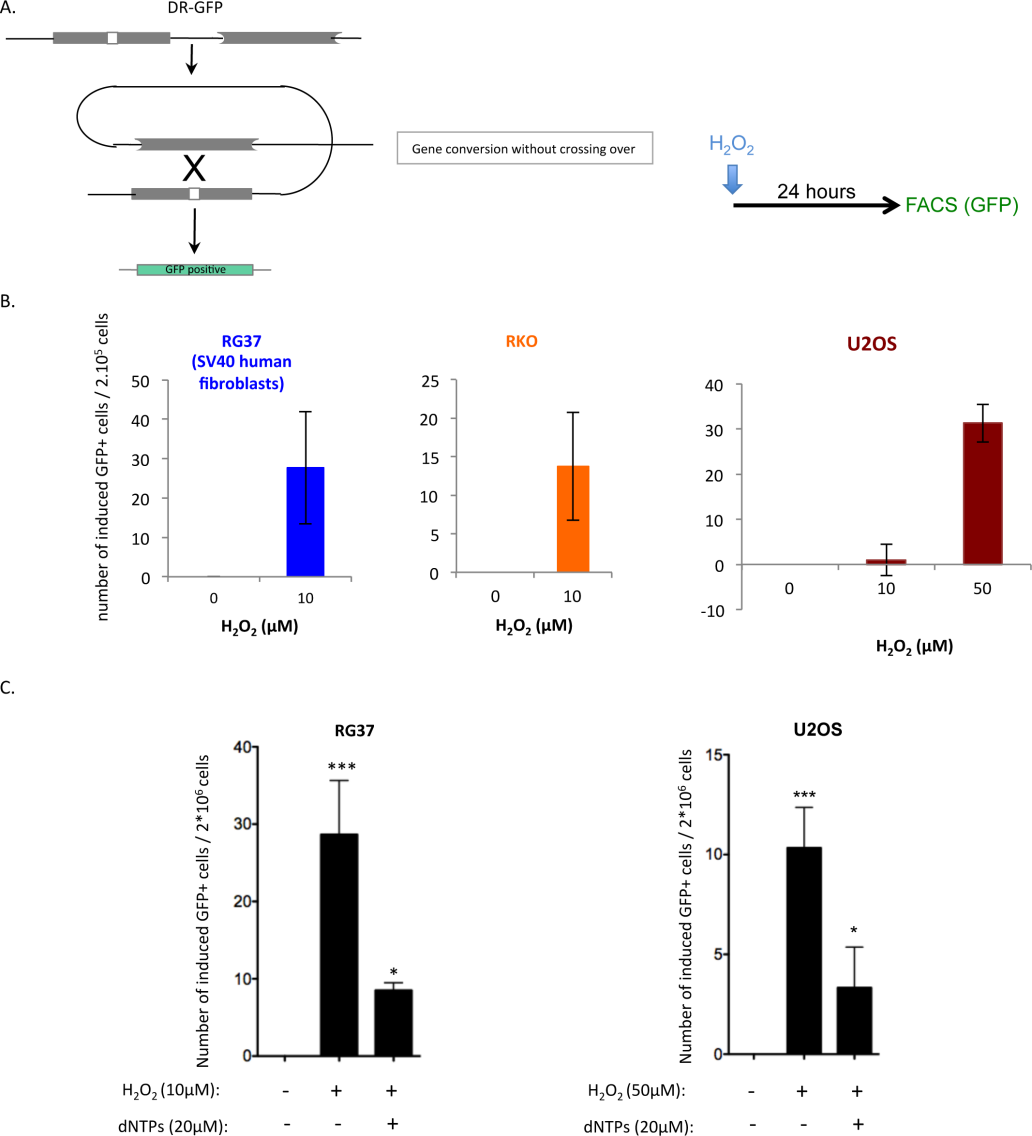
Impact of H_2_O_2_ on HR frequency. **A/** HR was measured in three different cell lines (U2OS, RKO and RG37, which is an SV40 immortalized human fibroblast [56]) bearing an intrachromosomal substrate (DR-GFP) that monitors HR [50]. Left panel: HR substrate (DR-GFP); two inactive GFP genes organized into direct repeats are integrated into the cells’ genomes. The 5’ GFP cassette is inactivated because of a mutational insertion (white scare). The 3’ GFP cassette is inactivated because of deletions in both the 5’ and 3’ sequences. HR between the two GFP genes can generate a functional GFP gene through gene conversion without crossing over. Recombinant cells are thus GFP-positive (GFP^+^) and can be monitored by FACS [50]. Right panel: experimental scheme. **B/** Induction of HR events by H_2_O_2_ in the RG37 (left panel), RKO (middle panel) and U2OS (right panel) cell lines. The values correspond to the induced number of recombinant cells (GFP^+^): the number of GFP^+^ cells in 2x10^5^ cells exposed to H_2_O_2_ subtracted from the number of GFP^+^ cell clones among 2x10^5^ untreated cells. The values correspond to at least three independent experiments. **C/** Impact of supplying dNs on H_2_O_2_-induced HR. The values correspond to at least three independent experiments.

To analyze the impact of OS on HR, we used 3 different human cell lines that bear intrachromosomal DR-GFP substrates (Fig 7A), which specifically monitor gene conversion [50].

Remarkably, 10 μM H_2_O_2_ stimulated HR (Fig 7B) in two different human cell lines (RG37 and RKO). Higher doses of H_2_O_2_ were too toxic to allow for HR monitoring. In the third human cell line (U2OS), HR stimulation was detectable upon exposure to 50μM H_2_O_2_ (Fig 7B). These data show that OS actually generates a recombinogenic stress.

Remarkably, supplying dNs partially decreased the OS-induced HR (Fig 7C). Because replication stress, which can be rescued by the dNTP supply, stimulates HR [49], these data suggest that OS-induced HR results from the replication stress generated by OS. Competition for dNTP uptake between DNA replication and DNA repair can account for these data, as discussed below.

## Discussion

HR plays a pivotal role in the reactivation of arrested replication forks. In addition, HR proteins, such as BRCA2 or RAD51, can also protect arrested RFs without leading to a recombination outcome [15–17]. Consequently, HR deficiency reveals the endogenous causes of spontaneous replication stress. Consistently, *HR*^*-*^ cells experience spontaneous genome-wide RF deceleration [4,18]. Here, we have identified endogenous OS as a major endogenous cause of the spontaneous RF deceleration that arises in *HR*^*-*^ cells. These data show that the two most frequently evoked endogenous stresses during oncogenesis and senescence initiation are linked and that defects in HR exacerbate the endogenous replication stress that is caused by endogenous OS.

The impact of OS on genetic instability through direct injury of the DNA, such as oxidized bases or abasic sites, which lead to local mutagenesis, has been documented extensively. In addition, the incorporation of oxidized nucleotides results in a pre-mutagenic lesion in the genome [51]. We show here an additional mechanism by which OS mediates genetic instability (i.e., chromosome mis-segregation through the formation of MECs generated by replication stress). Indeed, altered replication dynamics ultimately lead to mitotic defects [4]. Here, we show that OS generates MECs, which is consistent with the impact of OS on replication kinetics. MECs are a potential source of multipolar segregation or more hazardous merotelic kinetochore attachments that escape the mitotic spindle checkpoint. Thus, in addition to point mutations in the DNA caused by oxidized bases, OS might lead to numerical aneuploidy due to the mis-segregation of whole chromosomes that results from replication problems.

Moreover, OS can affect replication through several parallel mechanisms. First, replication stress can be generated by OS-induced DNA damage. Indeed, some types of oxidized bases can block the progression of polymerases [20], and OS-induced DSBs can lead to RF collapse. Second, the OS-induced oxidation of proteins involved in replication may alter replication initiation [21–26]. Third, we show here that the redox status of the cells strongly affects the dNTP pool.

The data presented here also reveal that the absence of HR exacerbates the replication stress that results from endogenous OS. First, the level of endogenous ROS is increased in *HR*^*-*^ cells. Cells defective in different DNA repair actors, such as ataxia telangiectasia, Fanconi anemia, and Cockayne and Bloom syndrome cells or BRCA1-defective cells, commonly show elevated levels of endogenous ROS [27–32]. Thus, the elucidation of the origin(s) of the elevated level of endogenous ROS in DNA repair-defective cells represents an exciting challenge for future studies. Remarkably, supplying dNs and treating the cells with anti-oxidants both restore the replication speed in *HR*^*-*^ cells without affecting the replication kinetics of WT cells (compare the present data with previous data [4]). Interestingly, NAC treatment increases the concentrations of all dNTPs, thus corresponding to a situation similar to the supply of exogenous dNs. In many different situations, supplying dNs rescues decelerated replication speed [4,40–43]. In particular, nucleotide deficiency has been proposed as a cause of genetic instability during the early steps of cancer development [41]. Therefore, it is tempting to speculate that anti-oxidant treatment might also recue replication and genome stability in these other situations.

ROS can alter replication through different mechanisms, including replication protein oxidation and oxidative damage to the DNA, leading to RF arrest. We show here that *HR*^*-*^ cells accumulate DNA damage that is rescued by anti-oxidant treatment. Such DNA damage accumulation in *HR*^*-*^ cells can result from several causes: 1- the increase of endogenous ROS in *HR*^*-*^ cells, as shown here, and 2- the accumulation of unrepaired recombinogenic damage. In addition, we show here that OS stimulates HR. However, because replication stress induces HR [49], the fact that OS-induced HR is rescued by supplying dNs suggests that in fact, enhanced HR results from the replication stress generated by the OS.

**Fig 8:**
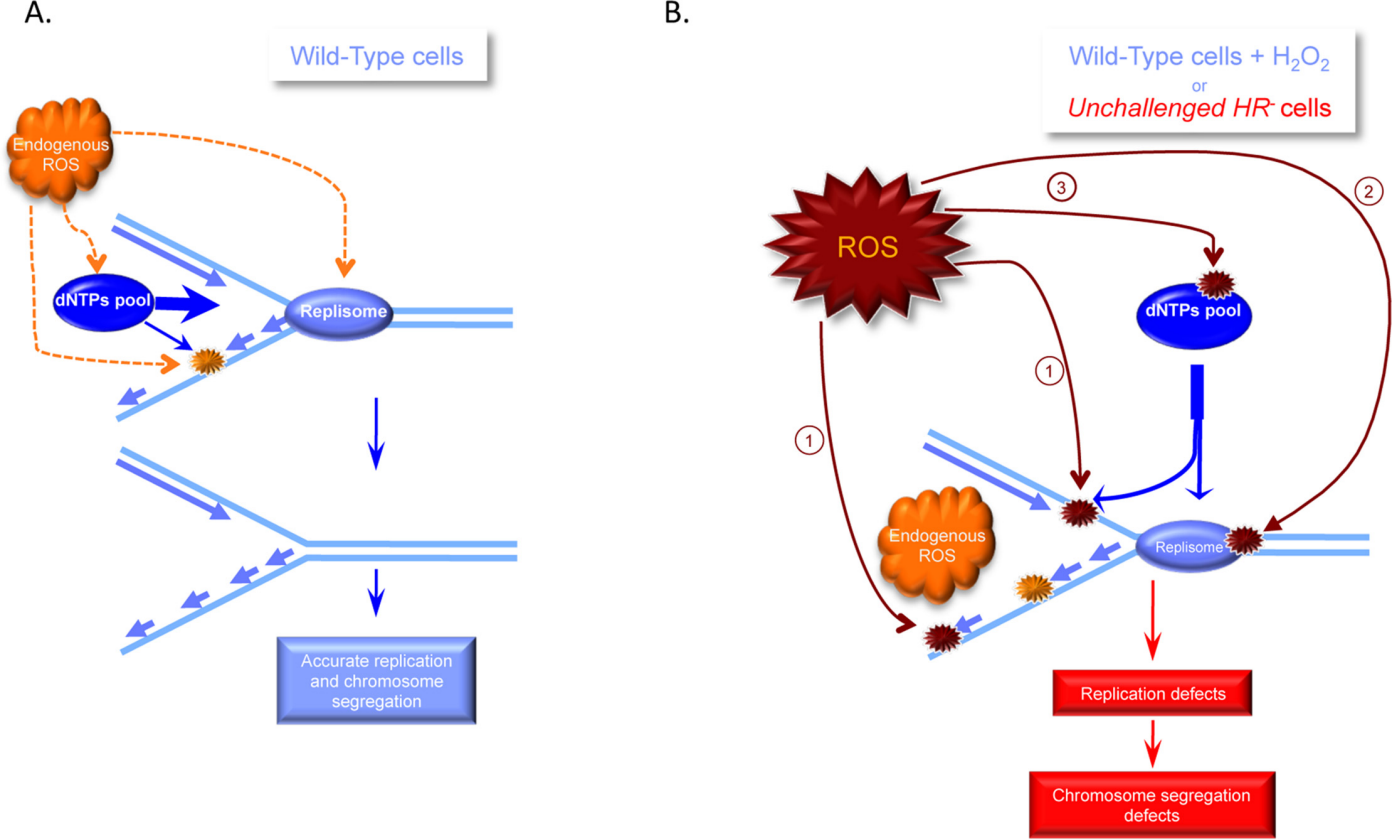
Impact of endogenous versus exogenous OS on replication. **A/** In WT cells, endogenous OS can produce spontaneous DNA damage, replisome alterations and dNTP pool restriction. However, the cells have reached a steady state that allows the delivery of dNTPs to DNA repair without affecting replication. **B/** An exogenous OS stress or HR defect increases the level of endogenous ROS, generating additional DNA damage (1) and altering replisome proteins (2) and the dNTP pool (3). In addition, the accumulation of DNA damage diverts dNTPs at the expense of replication. All of these processes affect replication dynamics, leading to chromosome segregation defects. Supplying dNs rescues both DNA repair and replication.

Collectively, these data can be unified as follows (Fig 8).

ROS can alter replication through different targets: DNA damage [20], replication proteins [21–26], and the nucleotide pool (present data). When maintained at 20% O_2_, WT cells have adapted to manage both the DNA damage and replication stress that result from endogenous OS. Indeed, neither maintaining unchallenged WT cells at low O_2_ concentrations nor treatment with NAC modified the replication fork kinetics of the cells. In addition, supplying dNs to unchallenged WT cells does not affect the replication speed [4]. However, transient exposure to exogenous OS alters the nucleotide pool, generates DNA damage and might alter replication proteins, which all affect RF progression. The nucleotide pool has been shown to be a limiting factor in yeast [38]. The fact that doses of HU as low as 10 μM, which only slightly alter the dNTP pool (see Fig 5), alter the replication speed [4] suggests that the dNTP pool is also a limiting factor in mammalian cells. However, the nucleotide pool is not expendable, even after genotoxic stress [52,53]. The accumulation of endogenous damage should then lead to the consumption of dNTPs for DNA repair at the expense of DNA replication, resulting in a dNTP shortage for replication and subsequently in replication stress, which should induce HR. Supplying nucleotides to OS-exposed WT cells abrogates the nucleotide shortage for replication, thus rescuing the replication speed and *in fine* normal HR levels. Importantly, under anti-oxidant conditions, the dNTP pool increases and therefore should not constitute a limiting factor. Similar to many other DNA repair-deficient cells, *HR*^*-*^ cells exhibit high levels of endogenous ROS, leading to the accumulation of DNA damage and an unbalanced dNTP pool. This scenario generates replication stress that ultimately leads to mitotic segregation defects [4]. Finally, supplying dNs suppresses the nucleotide shortage for replication, thus rescuing the replication speed. Antioxidant treatments, which decrease DNA damage in parallel, also increase the nucleotide pool and should thus suppress the dNTP shortage for replication. These situations rescue both the replication speed and the uneven centrosome duplication that occurs during mitosis. It is tempting to speculate that other endogenous stresses that may also alter replication, such as RNA/DNA hybrids, lead to a similar scenario.

Endogenous stress is a challenge that is encountered by cells daily, which can lead to genetic instability and tumor development or senescence initiation/progression. Here, we show that two major endogenous stresses are linked because endogenous OS is able to provoke spontaneous replication stress, leading to the formation of extra centrosomes during mitosis and subsequent aneuploidy. The present data highlight the importance of HR in protecting against the chromosome instability that is generated by endogenous oxidative stress.

## Materials and Methods

### Cell lines and treatments

The cell lines that were used in this study were derived from hamster V79 cells because several extensively characterized *HR*^*-*^ mutants exist in this background. V79SM24 is a V79-derivative cell line that stably expresses the dominant-negative RAD51 SMRAD51; V79-puro cells are V79 cells that are stably transfected with empty expression vectors [36,37]. We also used the V-C8 cell line, which is defective in the BRCA2 gene, and its counterpart V-C8#13, in which BRCA2 is complemented by human chromosome 13 [33]. V-C8#13 cells corresponded to a second WT cell line. Additionally, replication velocity has been widely analyzed in these cell lines [4,18].

The cell lines were cultured at 37°C under 5% CO_2_ in MEM supplemented with 10% fetal bovine serum, 2 mM glutamine, 200 IU/ml of penicillin and 200 μg/ml of streptomycin.

The cells were exposed to H_2_O_2_ or NAC for 48 hours (fresh H_2_O_2_ or NAC were added every 24 hours at the indicated doses). The cells were maintained under 3% oxygen for several weeks prior to the analyses.

Nucleotide precursors were supplied by adding a mixture of the four dNs (20 μM each) for 48 h. Deoxycytidine (Sigma D0776) was solubilized in 1 M NaOH (100 mM). Deoxyadenosine (Sigma D8668) was solubilized in 0.1 M NaOH (20 mM). Thymidine (Sigma T1895) was solubilized in H_2_O (50 mM). Deoxyguanosine (Sigma D7145) was solubilized in 1 M NH_4_OH (100 mM).

### Molecular combing

Molecular combing was performed as described previously [54]. IdU and CldU labeling (20 min pulse labeling each) were performed as described previously [40]. The analogs were revealed by incubating coverslips for 1 hour with a 1/5 dilution of a mouse anti-BrdU FITC antibody (Becton Dickson) and a 1/25 dilution of a rat anti-BrdU antibody (Seralab). After washing with 0.5 M NaCl, 20 mM Tris pH 7.8 and 0.05% Tween, the coverslips were incubated with secondary antibodies, including a 1/50 dilution of an Alexa Fluor 488-conjugated goat anti-mouse antibody (Molecular Probes) and a 1/50 dilution of an Alexa Fluor 594-conjugated goat anti-rat antibody (Molecular Probes). After washing, the coverslips were incubated sequentially with three antibodies, including a 1/100 dilution of a mouse anti-DNA antibody (Argene Biosoft), a 1/50 dilution of a rabbit anti-mouse Alexa 350 antibody (Molecular Probes) and a 1/25 dilution of a goat anti-rabbit Alexa 350 antibody (Molecular Probes). One hundred fifty fibers with symmetrical green-red labeling were analyzed for each cell line. The images were obtained and processed as described previously [40]. To focus on replication kinetics, we focused here on symmetrically double-labeled fibers, as described previously [4,40,55].

### Centrosome analysis

The cells were fixed in methanol for 15 min at –20°C and were then permeabilized with acetone. After three washes with PBS, the cells were blocked with 2% bovine serum albumin for 30 min and washed three times with PBS. The centrosomes were then stained with a γ-tubulin antibody (Sigma) that was diluted 1/200 in 0.5% BSA and 0.05% Tween for 1 hour at 37°C. The cells were washed three times with 0.05% Tween in PBS and incubated with an anti-rabbit antibody that was coupled to cyanine 2 (Jackson) and diluted 1/300 in 0.5% BSA and 0.05% Tween for 1 hour at 37°C. After three washes with 0.05% Tween in PBS, the cells were incubated with DAPI (5 μg/ml). For each cell line, 200 metaphase cells were analyzed.

### ROS measurement

Cellular ROS production was measured using a DCFDA assay kit according to the recommendations of the manufacturer. DCFDA (2’,7’-dichlorofluorescein diacetate), which is a cell-permeable fluorogenic dye, is deacetylated to a non-fluorescent compound by cellular esterases and later oxidized by ROS into the highly fluorescent 2’,7’-dichlorofluorescein (DCF), which measures hydroxyl, peroxyl and other ROS activity within the cell. A total of 10^5^ cells/well were plated in 6-well plates and cultured at 37°C in 5% CO_2_. After 2 days, the cells were rinsed in PBS, then incubated in 10 μM CM-H2DCF-DA (Life Technologies, USA) in DMEM with 1% FBS (Ex: 495 nm, Em: 520 nm) at 37°C for 45 min in the dark. The cells were trypsinized and re-suspended in DMEM with 1% FBS. The pelleted cells were washed again, and the pelleted live cells were re-suspended in PBS and analyzed using a BD Accuri C6 flow cytometer (BD Biosciences, San Diego, CA) with an FL1 laser (515–545 nm). The data are presented as the mean percentage of four independent experiments. Relative changes in DCF fluorescence were expressed as the fold increase in comparison to untreated cells.

### HR measurement

The cells were exposed to H_2_O_2_ at the indicated doses for 48 hours. For each data point, 2x 10^5^ cells were analyzed by FACS to monitor GFP-positive cells.

### Nucleotide quantification

The cells were washed with phosphate buffered saline (PBS), and the nucleotides were extracted with 3 ml of a mixture containing methanol and water (70/30; v/v). The extract was transferred into a tube, shaken for 30 s, and frozen at -80°C for further analyses. On the day of the analysis, the samples were vortexed vigorously after the addition of labeled calibration standard (2’-deoxyadenosine-^13^C_10_,^15^N_5_ 5’-triphosphate, 2’-deoxycytidine-^13^C_9_,^15^N_3_ 5’-triphosphate, 2’-deoxyguanosine-^13^C_10_,^15^N_5_ 5’-triphosphate and thymidine-^13^C_10_,^15^N_2_ 5’-triphosphate) and labeled internal standard (adenosine-^13^C_10_ 5’-triphosphate and cytidine-^13^C_9_ 5’-triphosphate) solutions. Next, the samples were centrifuged for 10 min at 15,000 g, and the supernatants were evaporated to dryness under nitrogen at 37°C. Finally, the residues were resuspended before injection into the liquid chromatography-tandem mass spectrometer. The analytical conditions used to quantify dATP, dGTP, dCTP and dTTP have been described previously [45]. The results for the quantification of the deoxynucleotides were normalized for one million cells.

### Western blotting

Non-reduced protein extracts (30 μg) were resolved using 8% SDS-PAGE, transferred to a nitrocellulose membrane, and probed with the following specific antibodies: anti-R1 (sc-11733, Santa Cruz), anti-R2 (sc-10844, Santa Cruz), and anti-tubulin (Sigma #T5168). Immunoreactivity was visualized using an enhanced chemiluminescence detection kit (Pierce ECL Western Blotting Substrate, Thermo Scientific).

### Immunofluorescence

The cells were grown on glass coverslips, fixed with 2% paraformaldehyde and permeabilized with 0.5% Triton-X100. After blocking, the cells were incubated with anti-XRCC1 (ab1838, Abcam, Inc.) or anti-phospho-Histone H2A.X (Ser139) primary antibodies (clone JBW301, Merck-Millipore, USA) diluted in PBS containing 1% BSA and 0.05% Tween. After washing with PBS containing 1% BSA, the cells were incubated with Alexa 488-conjugated anti-mouse secondary antibodies (Invitrogen, Molecular Probes) and stained with DAPI. Images were captured using a Zeiss motorized Axio Imager Z2 epifluorescence microscope with a ×63/1.4 NA oil immersion objective equipped with a Hamamatsu camera. Data acquisition was performed using AxioVision (4.7.2.). The number of foci per nucleus was measured with the Image J software.

### Statistical analyses

The distributions of RF velocity and origin firing in HR^-^ and WT cells were compared using the Mann-Whitney test.

## Supporting Information

S1 Data Replication speed in human lymphoblastoid (JEFF) cells upon silencing of RAD51. (DOCX)

S2 Data Survival after exposure to 10 μM H_2_O_2_. (DOCX)

S3 Data Redox status of RRM2 after exposure to H_2_O_2_ and in HR- cells. (DOCX)

## References

[1] Gelot C, Magdalou I, Lopez BS. Replication stress in Mammalian cells and its consequences for mitosis. Genes (Basel). 2015;6: 267–98. 10.3390/genes6020267 26010955 PMC4488665

[2] Magdalou I, Lopez BS, Pasero P, Lambert SA. The causes of replication stress and their consequences on genome stability and cell fate. [Internet]. Semin Cell Dev Biol. 2014 pp. 154–164. 10.1016/j.semcdb.2014.04.035 24818779

[3] Debatisse M, Le Tallec B, Letessier A, Dutrillaux B, Brison O. Common fragile sites: mechanisms of instability revisited. Trends Genet. 2012 pp. 22–32. 10.1016/j.tig.2011.10.003 22094264

[4] Wilhelm T, Magdalou I, Barascu A, Techer H, Debatisse M, Lopez BS. Spontaneous slow replication fork progression elicits mitosis alterations in homologous recombination-deficient mammalian cells. Proc Natl Acad Sci U S A. 2014 pp. 763–768. 10.1073/pnas.1311520111 24347643 PMC3896206

[5] Bartkova J, Horejsi Z, Koed K, Kramer A, Tort F, Zieger K, et al DNA damage response as a candidate anti-cancer barrier in early human tumorigenesis. Nature. 2005 pp. 864–870. 15829956 10.1038/nature03482

[6] Di Micco R, Fumagalli M, Cicalese A, Piccinin S, Gasparini P, Luise C, et al Oncogene-induced senescence is a DNA damage response triggered by DNA hyper-replication. Nature. 2006 pp. 638–642. 10.1038/nature05327 17136094

[7] Gorgoulis VG, Vassiliou L V, Karakaidos P, Zacharatos P, Kotsinas A, Liloglou T, et al Activation of the DNA damage checkpoint and genomic instability in human precancerous lesions. Nature. 2005 pp. 907–913. 15829965 10.1038/nature03485

[8] Gorgoulis VG, Halazonetis TD. Oncogene-induced senescence: the bright and dark side of the response. Curr Opin Cell Biol. 2010 pp. 816–827. 10.1016/j.ceb.2010.07.013 20807678

[9] Kramer A. Centrosome aberrations—hen or egg in cancer initiation and progression? Leukemia. 2005 pp. 1142–1144. 15858612 10.1038/sj.leu.2403780

[10] Nigg EA. Centrosome aberrations: cause or consequence of cancer progression? Nat Rev Cancer. 2002 pp. 815–825. 12415252 10.1038/nrc924

[11] Sluder G, Nordberg JJ. The good, the bad and the ugly: the practical consequences of centrosome amplification. Curr Opin Cell Biol. 2004 pp. 49–54. 15037304 10.1016/j.ceb.2003.11.006

[12] Boveri T. Zur Frage der Entstehung maligner Tumoren. Jena: Gustav Fisher 1914.

[13] Boveri T. Concerning the origin of malignant tumours by Theodor Boveri. Translated and annotated by Henry Harris. J Cell Sci. 2008 pp. 1–84. 10.1242/jcs.025742 18089652

[14] Costes A, Lambert SA. Homologous Recombination as a Replication Fork Escort: Fork-Protection and Recovery. Biomolecules,. 2012 pp. 39–71. 10.3390/biom3010039 24970156 PMC4030885

[15] Hashimoto Y, Chaudhuri AR, Lopes M, Costanzo V. Rad51 protects nascent DNA from Mre11-dependent degradation and promotes continuous DNA synthesis. Nat Struct Mol Biol. 2010. 10.1038/nsmb.1927 PMC4306207 20935632

[16] Schlacher K, Christ N, Siaud N, Egashira A, Wu H, Jasin M. Double-strand break repair-independent role for BRCA2 in blocking stalled replication fork degradation by MRE11 Cell. 2011 pp. 529–542. 10.1016/j.cell.2011.03.041 21565612 PMC3261725

[17] Ying S, Hamdy FC, Helleday T. Mre11-dependent degradation of stalled DNA replication forks is prevented by BRCA2 and PARP1. Cancer Res. 2012;72: 2814–2821. 10.1158/0008-5472.CAN-11-3417 22447567

[18] Daboussi F, Courbet S, Benhamou S, Kannouche P, Zdzienicka MZ, Debatisse M, et al A homologous recombination defect affects replication-fork progression in mammalian cells. J Cell Sci. 2008 pp. 162–166. 18089650 10.1242/jcs.010330

[19] Hyrien O. Mechanisms and consequences of replication fork arrest. Biochimie. 2000 pp. 5–17. 10717381 10.1016/s0300-9084(00)00344-8

[20] Wallace SS. Biological consequences of free radical-damaged DNA bases. Free Radic Biol Med. 2002 pp. 1–14. 12086677 10.1016/s0891-5849(02)00827-4

[21] Girard PM, Pozzebon M, Delacote F, Douki T, Smirnova V, Sage E. Inhibition of S-phase progression triggered by UVA-induced ROS does not require a functional DNA damage checkpoint response in mammalian cells. DNA Repair (Amst). 2008 pp. 1500–1516. 18603484 10.1016/j.dnarep.2008.05.004

[22] Montaner B, O’Donovan P, Reelfs O, Perrett CM, Zhang X, Xu YZ, et al Reactive oxygen-mediated damage to a human DNA replication and repair protein. EMBO Rep. 2007 pp. 1074–1079. 17932513 10.1038/sj.embor.7401084 PMC2247395

[23] Onn I, Milman-Shtepel N, Shlomai J. Redox potential regulates binding of universal minicircle sequence binding protein at the kinetoplast DNA replication origin. Eukaryot Cell. United States; 2004 pp. 277–287. 15075258 10.1128/EC.3.2.277-287.2004 PMC387648

[24] Sanders CM, Sizov D, Seavers PR, Ortiz-Lombardia M, Antson AA. Transcription activator structure reveals redox control of a replication initiation reaction. Nucleic Acids Res. England; 2007 pp. 3504–3515. 17478495 10.1093/nar/gkm166 PMC1904295

[25] Wang M, You JS, Lee SH. Role of zinc-finger motif in redox regulation of human replication protein A. Antioxid Redox Signal. United States; 2001 pp. 657–669. 11554452 10.1089/15230860152543005

[26] Weiner BE, Huang H, Dattilo BM, Nilges MJ, Fanning E, Chazin WJ. An iron-sulfur cluster in the C-terminal domain of the p58 subunit of human DNA primase. J Biol Chem. United States; 2007 pp. 33444–33451. 17893144 10.1074/jbc.M705826200

[27] Gorrini C, Baniasadi PS, Harris IS, Silvester J, Inoue S, Snow B, et al BRCA1 interacts with Nrf2 to regulate antioxidant signaling and cell survival. J Exp Med. 2013;210: 1529–44. 10.1084/jem.20121337 23857982 PMC3727320

[28] Barascu A, Le Chalony C, Pennarun G, Genet D, Imam N, Lopez B, et al Oxidative stress induces an ATM-independent senescence pathway through p38 MAPK-mediated lamin B1 accumulation. EMBO J. 2012;31: 1080–1094. 10.1038/emboj.2011.492 22246186 PMC3297999

[29] Zatterale A, Kelly FJ, Degan P, d’Ischia M, Pallard F V., Calzone R, et al Oxidative stress biomarkers in four Bloom syndrome (BS) patients and in their parents suggest in vivo redox abnormalities in BS phenotype. Clin Biochem. 2007;40: 1100–1103. 17678887 10.1016/j.clinbiochem.2007.06.003

[30] Andrade LN, Nathanson JL, Yeo GW, Menck CF, Muotri AR. Evidence for premature aging due to oxidative stress in iPSCs from Cockayne syndrome Hum Mol Genet. pp. 3825–3834. 10.1093/hmg/dds211 22661500 PMC3412382

[31] Pagano G, Talamanca AA, Castello G, Pallardo F V, Zatterale A, Degan P. Oxidative stress in Fanconi anaemia: from cells and molecules towards prospects in clinical management Biol Chem. pp. 11–21. 10.1515/BC-2011-227 22628295

[32] Zhang X, Sejas DP, Qiu Y, Williams D a, Pang Q. Inflammatory ROS promote and cooperate with the Fanconi anemia mutation for hematopoietic senescence. J Cell Sci. 2007;120: 1572–1583. 17405815 10.1242/jcs.003152 PMC2857731

[33] Kraakman-van der Zwet M, Overkamp WJ, van Lange RE, Essers J, van Duijn-Goedhart A, Wiggers I, et al Brca2 (XRCC11) deficiency results in radioresistant DNA synthesis and a higher frequency of spontaneous deletions. Mol Cell Biol. 2002 pp. 669–679. 11756561 10.1128/MCB.22.2.669-679.2002 PMC139737

[34] Wiegant WW, Overmeer RM, Godthelp BC, van Buul PP, Zdzienicka MZ. Chinese hamster cell mutant, V-C8, a model for analysis of Brca2 function. Mutat Res. 2006 pp. 79–88. 10.1016/j.mrfmmm.2006.03.001 16643964

[35] Bertrand P, Lambert S, Joubert C, Lopez BS. Overexpression of mammalian Rad51 does not stimulate tumorigenesis while a dominant-negative Rad51 affects centrosome fragmentation, ploidy and stimulates tumorigenesis, in p53-defective CHO cells. Oncogene. 2003 pp. 7587–7592. 14576820 10.1038/sj.onc.1206998

[36] Lambert S, Lopez BS. Characterization of mammalian RAD51 double strand break repair using non lethal dominant negative forms. EMBO J. 2000 pp. 3090–3099. 10856252 10.1093/emboj/19.12.3090 PMC203369

[37] Daboussi F, Thacker J, Lopez BS. Genetic interactions between RAD51 and its paralogues for centrosome fragmentation and ploidy control, independently of the sensitivity to genotoxic stresses. Oncogene. 2005 pp. 3691–3696. 15782136 10.1038/sj.onc.1208438

[38] Das KC, Dashnamoorthy R. Hyperoxia activates the ATR-Chk1 pathway and phosphorylates p53 at multiple sites. Am J Physiol Lung Cell Mol Physiol. 2004 pp. L87–97. 12959929 10.1152/ajplung.00203.2002

[39] Willis J, Patel Y, Lentz BL, Yan S. APE2 is required for ATR-Chk1 checkpoint activation in response to oxidative stress. Proc Natl Acad Sci U S A. 2013 pp. 10592–10597. 10.1073/pnas.1301445110 23754435 PMC3696815

[40] Anglana M, Apiou F, Bensimon A, Debatisse M. Dynamics of DNA replication in mammalian somatic cells: nucleotide pool modulates origin choice and interorigin spacing. Cell. 2003 pp. 385–394. 12914702 10.1016/s0092-8674(03)00569-5

[41] Bester AC, Roniger M, Oren YS, Im MM, Sarni D, Chaoat M, et al Nucleotide deficiency promotes genomic instability in early stages of cancer development. Cell. United States; 2011 pp. 435–446. 10.1016/j.cell.2011.03.044 PMC3740329 21529715

[42] Chabosseau P, Buhagiar-Labarchede G, Onclercq-Delic R, Lambert S, Debatisse M, Brison O, et al Pyrimidine pool imbalance induced by BLM helicase deficiency contributes to genetic instability in Bloom syndrome. Nat Commun. England; 2011 p. 368 10.1038/ncomms1363 21712816

[43] Gay S, Lachages AM, Millot GA, Courbet S, Letessier A, Debatisse M, et al Nucleotide supply, not local histone acetylation, sets replication origin usage in transcribed regions. EMBO Rep. England; 2010 pp. 698–704. 10.1038/embor.2010.112 20671737 PMC2933864

[44] Ma E, Goldar A, Verbavatz JM, Marsolier-Kergoat MC. Giant yeast cells with nonrecyclable ribonucleotide reductase. Mol Genet Genomics. 2011 pp. 415–425. 10.1007/s00438-011-0613-4 21442328

[45] Machon C, Jordheim LP, Puy JY, Lefebvre I, Dumontet C, Guitton J. Fully validated assay for the quantification of endogenous nucleoside mono- and triphosphates using online extraction coupled with liquid chromatography-tandem mass spectrometry. Anal Bioanal Chem. 2014 pp. 2925–2941. 10.1007/s00216-014-7711-1 24633509

[46] Poli J, Tsaponina O, Crabbe L, Keszthelyi A, Pantesco V, Chabes A, et al dNTP pools determine fork progression and origin usage under replication stress. EMBO J. England; 2012 pp. 883–894. 10.1038/emboj.2011.470 22234185 PMC3280562

[47] Fridlich R, Annamalai D, Roy R, Bernheim G, Powell SN. BRCA1 and BRCA2 protect against oxidative DNA damage converted into double-strand breaks during DNA replication. DNA Repair (Amst). 2015;30: 11–20. 10.1016/j.dnarep.2015.03.002 25836596 PMC4442488

[48] Liang F, Han M, Romanienko PJ, Jasin M. Homology-directed repair is a major double-strand break repair pathway in mammalian cells. Proc Natl Acad Sci U S A. 1998 pp. 5172–5177. 9560248 10.1073/pnas.95.9.5172 PMC20233

[49] Saintigny Y, Delacote F, Vares G, Petitot F, Lambert S, Averbeck D, et al Characterization of homologous recombination induced by replication inhibition in mammalian cells. EMBO J. 2001 pp. 3861–3870. 11447127 10.1093/emboj/20.14.3861 PMC125539

[50] Pierce AJ, Johnson RD, Thompson LH, Jasin M. XRCC3 promotes homology-directed repair of DNA damage in mammalian cells. Genes Dev. 1999 pp. 2633–2638. 10541549 10.1101/gad.13.20.2633 PMC317094

[51] Freudenthal BD, Beard WA, Perera L, Shock DD, Kim T, Schlick T, et al Uncovering the polymerase-induced cytotoxicity of an oxidized nucleotide. Nature. 2015;517: 635–639. 10.1038/nature13886 25409153 PMC4312183

[52] Niida H, Shimada M, Murakami H, Nakanishi M. Mechanisms of dNTP supply that play an essential role in maintaining genome integrity in eukaryotic cells. Cancer Sci. England; 2010 pp. 2505–2509. 10.1111/j.1349-7006.2010.01719.x 20874841 PMC11158391

[53] Hakansson P, Hofer A, Thelander L. Regulation of mammalian ribonucleotide reduction and dNTP pools after DNA damage and in resting cells. J Biol Chem. United States; 2006 pp. 7834–7841. 16436374 10.1074/jbc.M512894200

[54] Michalet X, Ekong R, Fougerousse F, Rousseaux S, Schurra C, Hornigold N, et al Dynamic molecular combing: stretching the whole human genome for high-resolution studies. Science. 1997 pp. 1518–1523. 9278517 10.1126/science.277.5331.1518

[55] Daboussi F, Courbet S, Benhamou S, Kannouche P, Zdzienicka MZ, Debatisse M, et al A homologous recombination defect affects replication-fork progression in mammalian cells. J Cell Sci. 2008;121: 162–166. 18089650 10.1242/jcs.010330

[56] Dumay A, Laulier C, Bertrand P, Saintigny Y, Lebrun F, Vayssiere JL, et al Bax and Bid, two proapoptotic Bcl-2 family members, inhibit homologous recombination, independently of apoptosis regulation. Oncogene. 2006 pp. 3196–3205. 16407825 10.1038/sj.onc.1209344

