# Supplementary material for: Slow Replication Fork Velocity of Homologous Recombination-Defective Cells Results from Endogenous Oxidative Stress
Source: PLoS Genet. 2016 May 2;12(5):e1006007. doi: 10.1371/journal.pgen.1006007 (PMC4852921; doi:10.1371/journal.pgen.1006007)
Supplement: S1 Data — (DOCX) [file pgen.1006007.s001.docx]

***Supplementary data***

***S1.* REPLICATION SPEED IN HUMAN LYMPHOBLASTOID (JEFF) CELLS UPON SILENCING OF RAD51.**

Silencing RAD51 leads to replication speed deceleration, which is rescued by supplying nucleotide precursors (dNs).


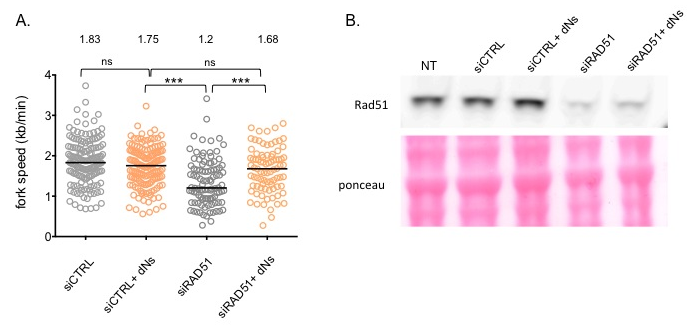


**Figure S1.** Dot-plots of the relative fork speed in JEFF cells silenced or not silenced for RAD51. The supplementation of dNs is indicated. The horizontal black lines represent the median of the relative fork speed distribution. The median values are indicated above the distributions. Right panels: Western blots showing the efficiency of RAD51-depletion in JEFF cells. Ponceau staining is used as a loading control.

**Cell culture and nucleotide precursor complementation.** JEFF cells were grown in RPMI 1640 + GlutaMAX-I medium (GIBCO) + 10% fetal calf serum and 10 μg/mL of penicillin and streptomycin (GIBCO). The medium was complemented with dNs (SIGMA) at a concentration of 20 μM each. siRNAs were transfected 48 h prior to analysis.
