# Supplementary material for: Slow Replication Fork Velocity of Homologous Recombination-Defective Cells Results from Endogenous Oxidative Stress
Source: PLoS Genet. 2016 May 2;12(5):e1006007. doi: 10.1371/journal.pgen.1006007 (PMC4852921; doi:10.1371/journal.pgen.1006007)
Supplement: S2 Data — (DOCX) [file pgen.1006007.s002.docx]

***Supplementary data***

***S2.* SURVIVAL AFTER EXPOSURE TO 10 µM H_2_O_2_.**

**
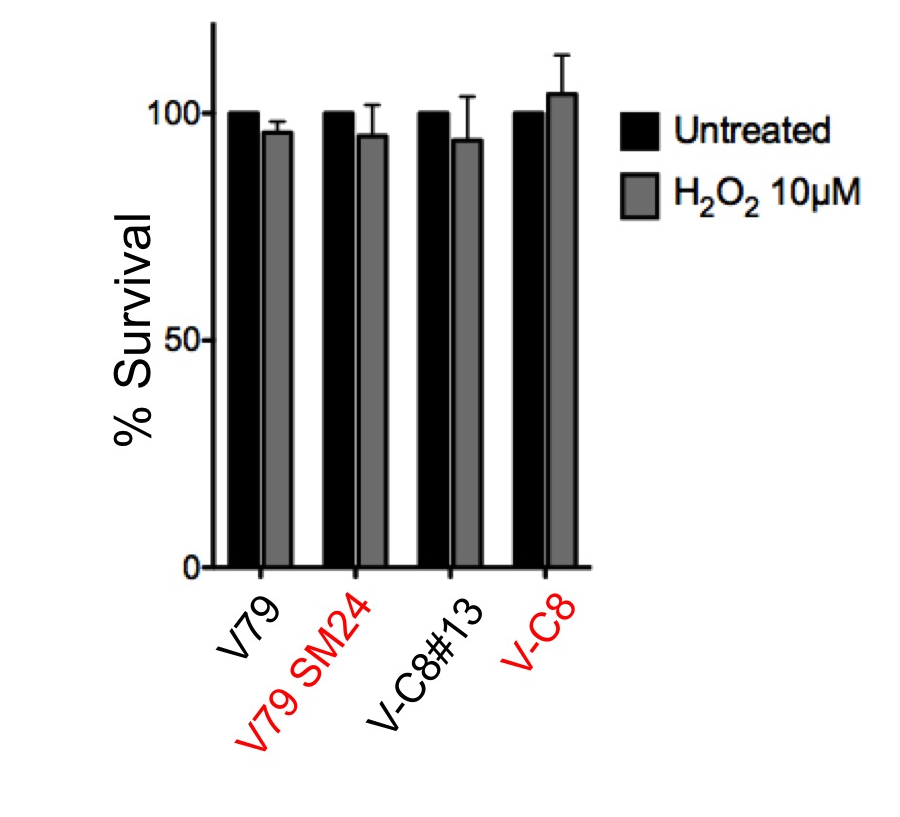
**

**Figure S2.** The cells were exposed to 10 µM HU for 48 h. Viability was measured by using the MTT assay. The values correspond to 3 independent experiments (with triplicate samples in each experiment).

**Cell viability measurement.** The cell viability of the cultures was determined by using the MTT assay. The cells were plated in 96-well culture plates and incubated with different concentrations of H_2_O_2_ for 48 h. After the treatments, the cells were incubated with medium and an MTT solution (1 mg/ml, prepared in a phosphate-buffered saline PBS solution) for 2-3 h at 37°C. Finally the medium was removed, and formazan particles were dissolved in dimethyl sulfoxide (DMSO). Cell viability, which was defined as the relative amount of MTT reduction, was determined via spectrophotometry at 570 nm.
