# Supplementary material for: Slow Replication Fork Velocity of Homologous Recombination-Defective Cells Results from Endogenous Oxidative Stress
Source: PLoS Genet. 2016 May 2;12(5):e1006007. doi: 10.1371/journal.pgen.1006007 (PMC4852921; doi:10.1371/journal.pgen.1006007)
Supplement: S3 Data — (DOCX) [file pgen.1006007.s003.docx]

***Supplementary data***

***S3.* REDOX STATUS OF RRM2 AFTER EXPOSURE TO H_2_O_2_ AND IN HR- CELLS.**


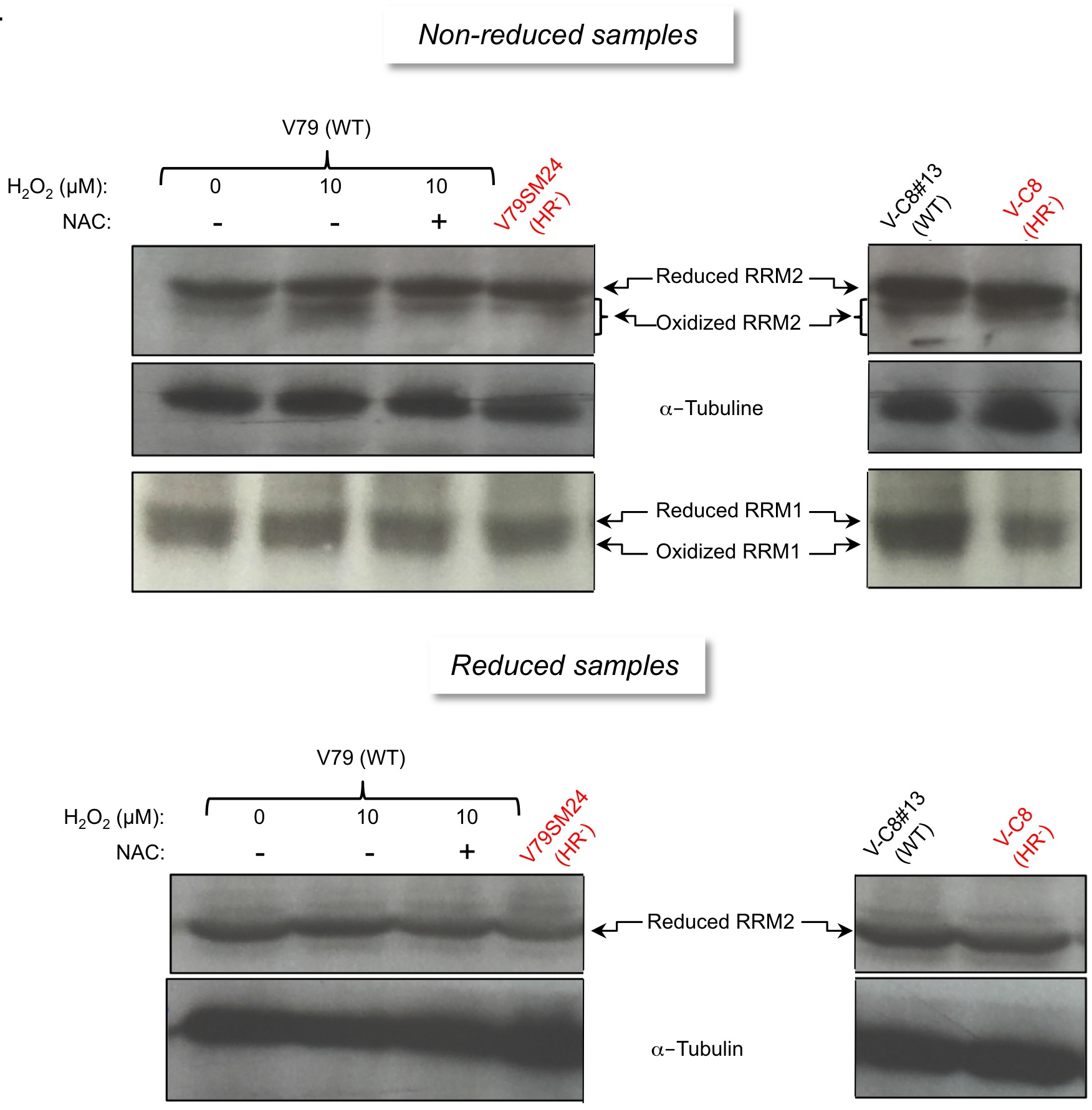


**Figures S3.** Western blot analysis of RRM2 or RRM1 after exposure to H_2_O_2_. Upper panel: electrophoresis of reduced samples. The samples were run on the same gel; after transfer, the membranes were cut for incubation with different specific antibodies. Lower panel: electrophoresis of non-reduced samples.

In the yeast *Saccharomyces cerevisiae*, the ribonucleotide reductase (RNR), which produces dNTPs, is oxidized by ROS [1,2]. Here, in mammalian cells, the electrophoresis of non-reduced samples revealed that 10 µM H_2_O_2_ was sufficient to slightly increase oxidized forms of the RRM2 subunit of the RNR but not of RRM1 (Figure S1). Interestingly, exposure to the anti-oxidant N-acetyl-cysteine (NAC) rescued the basal ratio of the reduced/oxidized forms of RRM2 (Figure S1). However, the redox status did not appear to be modified in HR- cells (V79SM24 and V-C8) in comparison to their respective controls. Surprisingly, despite the chronically higher levels of endogenous ROS, the endogenous redox status of RRM2 did not appear to be affected significantly in the *HR^-^* cells (due to the sensitivity of the method). Thus, RRM2 may have adapted to the chronically high levels of ROS in *HR^-^* cells.

**Reference.**

1. Camier S, Ma E, Leroy C, Pruvost A, Toledano M, Marsolier-Kergoat MC. Visualization of ribonucleotide reductase catalytic oxidation establishes thioredoxins as its major reductants in yeast. Free Radic Biol Med. 2007. pp. 1008–1016.

2. Ma E, Goldar A, Verbavatz JM, Marsolier-Kergoat MC. Giant yeast cells with nonrecyclable ribonucleotide reductase. Mol Genet Genomics. 2011. pp. 415–425.
